# Spinal anaesthesia for orthopaedic surgery in children with cerebral palsy: Analysis of 36 patients

**DOI:** 10.12669/pjms.311.5709

**Published:** 2015

**Authors:** Ozkan Onal, Seza Apiliogullari, Ergun Gunduz, Jale Bengi Celik, Hakan Senaran

**Affiliations:** 1 Ozkan Onal, Department of Anaesthesia and Intensive Care, Selcuk University Medical Faculty, Konya, Turkey.; 2 Seza Apiliogullari, Department of Anaesthesia and Intensive Care, Selcuk University Medical Faculty, Konya, Turkey.; 3 Ergun Gunduz, Department of Anaesthesia and Intensive Care, Selcuk University Medical Faculty, Konya, Turkey.; 4 Jale Bengi Celik, Department of Anaesthesia and Intensive Care, Selcuk University Medical Faculty, Konya, Turkey.; 5 Hakan Senaran, Department of Orthopaedics and Traumatology, Selcuk University Medical Faculty, Konya, Turkey.

**Keywords:** Spinal anesthesia, Cerebral palsy, Pediatric, Orthopaedic Surgery

## Abstract

**Background and Objective:**

Cerebral palsy is one of the most common childhood neuromuscular diseases in the world. Spinal anaesthesia in children is an evolving technique with many advantages in perioperative management. The aim of this retrospective study was to provide first-hand reports of children with cerebral palsy who underwent orthopaedic surgery under spinal anaesthesia.

**Methods:**

Records of the children with cerebral palsy who underwent orthopaedic surgery under spinal anaesthesia between May 2012 and June 2013 at Selcuk University Hospital were investigated. In all patients, lumbar puncture was performed in lateral decubitus position with mask sevoflurane-nitrous oxide anaesthesia. In patients who were calm prior the spinal block, inhalation anaesthesia was terminated. In patients who were restless before the spinal block, anaesthesia was combined with light sevoflurane anaesthesia and a laryngeal mask. From anaesthesia records, the number of attempts required to complete the lumbar puncture, and the success rates of spinal anaesthesia and perioperative complications were noted. Data were expressed as numbers and percentages.

**Results:**

The study included 36 patients (20 girls and 16 boys). The mean age was 71 months. The rate of reaching subarachnoid space on first attempt was 86%. In all patients, spinal anaesthesia was considered successful. In 26 patients, laryngeal mask and light sevoflurane anaesthesia were required to maintain ideal surgical conditions. No major perioperative complications were observed.

**Conclusion:**

Spinal anaesthesia alone or combined with light sevoflurane anaesthesia is a reliable technique with high success rates in children with cerebral palsy undergoing orthopaedic surgery.

## INTRODUCTION

Cerebral palsy (CP) affects 1:500 children globally, making it one of the world’s most common neuromuscular diseases.^1^^,^^2^ CP non-progressive neurological disorders of motor development in children are secondary to lesions or anomalies of the brain.^3^ Among children with CP, 61% undergo orthopaedic lower limb surgery of bone and soft tissues to improve mobility.^4^ Patients with CP present a wide range of clinical implications for the anaesthesiologist, because the clinical presentation can range from mild monoplegia with normal cognitive capacity to spastic quadriplegia with severe mental retardation.^5^ Therefore, each patient requires special anaesthetic considerations to accommodate his or her disabilities. Up to date, general anaesthesia was used in most of the paediatric CP case; however, in some of the selected cases either regional anaesthesia or combination of regional and general anaesthesia was applied.^6^^-^^9^

Within the last several years, regional anaesthesia in children has earned widespread approval. Today, it is a valid and effective technique used in the daily practices of many paediatric centres.^10^ Spinal anaesthesia (SA) in children has many advantages, including rapid onset and a profound and uniformly distributed sensory and motor block with a high success rate. The procedure also results in greater control of cardiovascular and stress responses compared to epidural or general anaesthesia in perioperative management.^11^^,^^12^ In the last 20 years, its popularity for paediatric patients has increased, and numerous investigations have been performed in both healthy and high-risk children.^13^^-^^16^ SA-related case reports are increasing in children with various neuromuscular disease,^14^^,^^17^ but to knowledge, there is no prospective or retrospective study investigating the appropriateness of SA in children with CP.

The aim of the present study was to present first-hand case reports of the success rate of SA in selected children with CP who underwent orthopaedic lower limb surgery.

## METHODS

The study was approved by the Research Ethics Committee of the Selcuk University Medical Faculty. For the present study, the anaesthesia form and special registry form of 36 children with CP in whom SA was attempted for lower limb surgery from May 2012 and June 2013 at Selcuk University Hospital were reviewed. The anaesthesiologist for each case undergoing SA completed a special registry form which comprised of the perioperative data. Cases with incomplete forms were excluded. 

Attending anaesthesiologist (S.A and O.O who experienced more than 500 and 50 pediatric spinal anesthesia respectively) performed a spinal block according to personal preference in selected children who were not taking antiepileptic medication and who had palpable interspace of the lumbar vertebrae. The paediatric spinal anaesthesia technique defined in the literature was used.^18^ After a pre-anaesthetic evaluation and parental consent, the patient was transported to the operating room. Monitors for ECG, non-invasive blood pressure, pulse oximetry and, if available, paediatric bispectral index sensors (BIS) were used. Measurements were recorded 5-minutes’ intervals. Active warming was started on the patient’s upper body using a forced-air warming system. Each child was sedated with 8% sevoflurane in a 60% N_2_O–40% O_2_ mixture during spontaneous breathing via a facemask. After establishing peripheral intravenous access, the child was placed in the lateral decubitus and, if possible, the knee-chest position with the table inclined to a 45-degree head-up tilt.^18^ An experienced anaesthesiologist performed the lumbar puncture with a midline approach using a 27G pencil point needle if available. If a 27G was not available, a 25G-quince needle was used. The most readily palpable interspace, S1 to L3 vertebrae, was chosen for the lumbar puncture. Correct placement of the needle was verified by a free flow of clear cerebrospinal fluid. Hyperbaric bupivacaine 0.5% was used for SA. The dose of bupivacaine was calculated according to the age of the child: < 5 year= 0.5 mg.kg^-1 ^and >5 year= 0.4mg.kg^-1^. The maximum dose of bupivacaine was 10 mg.

In children who were calm prior the spinal block, inhalation anaesthesia was terminated. In children who were restless before spinal block, anaesthesia was maintained with light sevoflurane anaesthesia and laryngeal mask airway insertion. The patient’s heart rate and arterial blood pressure were maintained within 20% of the preoperative values. To provide this condition, the concentration of sevoflurane was decreased to maintain a level of 0.7 minimum alveolar concentration (MAC). No other anaesthetics, such as neuromuscular blockade, analgesics or sedatives were administered. Hypotension, defined as a reduction of systolic blood pressure more than 20% from the baseline and bradycardia (<60 heart rate) were considered as the main intraoperative complication.

After surgical incision, if the patient’s heart rate and arterial blood pressure were increased more than 20%, concentration of sevoflurane was increased and fentanyl 2 mg.kg^-1^was applied intravenously. Following the operation, the child was transferred to the post-anaesthesia care unit (PACU) for continuous monitoring of vital signs at least one hour and pain management in the presence of their parents. Children were discharged from the PACU when they were able to move any part of their legs that considered as the main postoperative complication, fully awake and stable hemodynamic and respiratory conditions were ascertained. Special attention was paid to any signs and symptoms of unexpected long duration motor block of legs.

The primary goal of this study was to determine the number of attempts required to successfully lumbar puncture children with CP and the success rate of SA in these children. Any redirection of the spinal needle before appearance of cerebrospinal fluid was classified as another attempt. To evaluate the success of spinal anaesthesia, the extremity movement with pinprick or surgical stimulus, or the increase in pulse rate of more than 20% and the addition of fentanyl were used. The following procedure-related parameters were assessed as a secondary aim from records: patient demographics; type of spinal needle; increase in the pulse rate of more than 20% after surgical incision; number of patient who experienced hypotension and bradycardia, tachycardia and hypertension after SA, addition of ephedrine and atropine and fentanyl use; and the number of patients who required a laryngeal mask and light sevoflurane anaesthesia to remain calm and number of patient cannot move at least finger of legs before the discharge from the PACU. Spinal-block related postoperative complications, including PDPH and backache, could not be evaluated due to patients’ cognitive dysfunction. Results are presented as number of cases, percentage or mean ± standard deviation (SD).

## RESULTS

The study included 36 children (20 girls and 16 boys, 21 of 36 were performed by S.A). The mean age was 71 months, with a range of 13–144 months. Patients’ demographics and study outcomes are presented in Table I and II respectively. The rate of reaching the subarachnoid space at first attempt was 86%, and the rate of patients requiring a third attempt was 5.6%. In all patients, spinal anaesthesia was considered successful. In 26 patients, laryngeal mask and light sevoflurane anaesthesia were required to keep the patient calm and maintain ideal surgical conditions. Paediatric BIS were available in 21 of 26 patients who required light sevoflurane anaesthesia. The mean data of BIS was 60.5 with a range of 55–67 after SA during the 0.7 MAC of sevoflurane. Two children developed transient hypotension which not necessary ephedrine use. All patient discharge from the PACU uneventfully.

## DISCUSSION

To the best of our knowledge this is the first study demonstrating that SA alone or combined with light sevoflurane anaesthesia is a reliable technique with high success rates in selected children with cerebral palsy who are undergoing orthopaedic operations by experienced practitioners.

In paediatric patients with neurologic disorders and scoliosis-like anatomic variations of the vertebrae, decisions about SA are complicated. Curvature of the spine often makes SA difficult to perform and may cause an unsuccessful spinal block. The first-attempt success rate of lumbar puncture under general anaesthesia is over 90% in paediatric patients without neurologic disease who are older than one year.^18^^,^^19^ In the present study, the rate of reaching the subarachnoid space on first attempt was 86% in 36 children selected for spinal block due to absence of anatomic variations of the lumbar vertebrae. The literature review revealed a lack of accessible information on the success rates of reaching subarachnoid space and SA in children with CP. The reported success rates of SA were similar to previous reports in children without CP.^18^^,^^19^

Anxiety is an important issue for children in regional block applications and during the operation, particularly because, even without CP, many young patients cannot communicate effectively. Prospective and retrospective safety studies support the notion that performing regional anaesthesia under general anaesthesia is a safe practice.^20^ However, some children are able to remain calm and tolerate regional block application and short-term surgical procedures without deep sedation or general anaesthesia.^21^^,^^22^ Before the regional block, anaesthesia can be induced by inhalation or intravenously in children with CP, similar to healthy children. Low-concentration inhalation anaesthetics^7^^,^^14^ and intravenous agents (e.g., propofol, clonidine, ketamine and dexmedetomidine)^17^ were applied for sedation in children during the surgical procedures under regional blocks. In our clinic, most of the regional blocks performed on children under inhalation anaesthesia consist of N_2_O and sevoflurane. This technique provides intravenous access, painless lumbar puncture and, if necessary, effective and easy application of sedation during the operation using a low concentration of sevoflurane.

MAC (the response to a noxious stimulus) is mediated through the spinal cord.^23^ Sevofluran concentration that related to MAC value is reduced in children with CP, analgesic use and additional caudal block application.^7^^,^^24^ The spinal block has a sedative effect,^25^ although the concentration of sevoflurane has not been determined in children with CP under SA. Kim et al.^7^ showed that combined caudal-general anaesthesia is a 36% decrease in sevoflurane concentration compared to general anaesthesia, while maintaining the BIS values in a range of 45-55 during orthopaedic surgery in children with CP. In regular clinical practice, 0.7 MAC of sevoflurane is used to keep calm in 26 of 36 children with CP and none of them required additional fentanyl use after spinal block. Future prospective investigations should evaluate the lowest MAC of sevoflurane in children with CP with a laryngeal mask-supported airway under SA.

In paediatric patients, it is common practice to administer caudal epidural analgesia along with general anaesthesia to decrease intraoperative inhalational anaesthetic requirements^4^^,^^7^ and postoperative pain.^5^ In the present study, spinal block as a primer anaesthetic technique and light sevoflurane anaesthesia were applied to keep children calm. Rapid onset is a major advantage of spinal anaesthesia compared to caudal block. Hence, a high inhalation anaesthetic concentration and IV opioid application were not necessary following the lumbar puncture. Further controlled studies are necessary to evaluate the advantages and disadvantages of spinal block and caudal block in children with CP receiving lower limb surgery.

**Table-I:** Patient data are presented as number of patients, mean (range) (n=36).

**Gender (female/male)**	**Age (months)**	**Weight (kg)**	**ASA I / II / III**	**Duration of surgery (min)**
20/16	71.17 (13-144)	17.75 (8-39)	0/30/6	55.9 (30-125)

ASA: American Society of Anesthesiologists physical status.

**Table-II:** Procedural data of the study. Data are presented as number of patients (%).

**Needle type**	
27G Pencil point 25G Cutting point	15 (41.7) 21 (58.3)
**Number of attempts for successful LP**	
1 2 3	31 (86.1) 3 (8.3) 2 (5.6)
**Success rates **	
Successful SA Unsuccessful SA	36 (100) 0 (0)
BIS	55-67 (60.5+3.1)

LP: Lumbar puncture; SA: Spinal anaesthesia; BIS: Bispectral index sensors.

The most important differences between paediatric SA and adult SA technique is absence of serious hypotension that required ephedrine use in paediatric group. In the present study absent of intraoperative ephedrine use show that this technique is also safe in paediatric patient with CP. 

A number of key questions must still be addressed to understand the development and maintenance of the optimum perioperative management of spinal anaesthesia in children with CP. First, researchers must determine the safest and most practical sedative agent for use before neuroaxial block and during surgery in children with CP. Second, the different sevoflurane concentration used in children with CP under SA. Third, researchers must discover which anaesthetic technique is best for children with CP: caudal anaesthesia, spinal anaesthesia or combined spinal-epidural anaesthesia. Finally, it must be determined whether there are negative long-term effects of neuroaxial anaesthesia on neuromuscular condition among children with CP.

There are several limitations to this study. First, the study is retrospective. In addition, spinal-block related postoperative complications, including PDPH and backache, could not be evaluated due to patients’ cognitive dysfunction, although special attention was paid to use 27G pencil point needle to reduce PDPH. Patients were selected by the attending anaesthesiologist in the presented study, so the sample does not reflect all paediatric patients with CP.

In conclusion, spinal anaesthesia alone or combined with light sevoflurane anaesthesia is a reliable technique in selected children with cerebral palsy undergoing orthopaedics operations by experienced practitioners. This type of anaesthesia should be used in children who are at high risk during general anaesthesia. Further controlled studies are necessary to clarify the optimum intra operative management around the spinal anaesthesia in children with CP.
